# Examination of the reliability and validity of a French adaptation of a graphic single-item of organizational identification

**DOI:** 10.3389/fpsyg.2026.1651105

**Published:** 2026-03-18

**Authors:** Martin Lauzier, François Durand

**Affiliations:** 1 Department of Industrial Relations, Université du Québec en Outaouais, Gatineau, QC, Canada; 2 Telfer School of Management, University of Ottawa, Ottawa, ON, Canada

**Keywords:** graphic single-item, organizational identification, reliability, test-retest, validity

## Abstract

This research presents results from two studies investigating the psychometric qualities of a graphic single-item measuring organizational identification (i.e., the individual’s perception of unity and feeling of being one with the organization in which they work). Conducted on two samples, the first study revealed the convergent validity of a single-item graphic scale, obtaining a positive correlation with a measure of the same construct but comprising multiple written items. This study also showed the incremental validity of the graphic single-item in explaining job satisfaction and work engagement over and above the variance explained by the multiple written items instrument. The second study replicated the results observed in the first study and established the test-retest reliability of the graphic single-item. Taken together, these results recognize the potential and qualities of the French adaptation of the graphic single-item and offer researchers and organizations a rapid and effective way of measuring this construct in French-speaking populations.

## Introduction

Organization identification (OI), defined as “the perception of oneness with or belongingness to an organization” (Mael and Ashforth, 1992, p. 104), is an important part of contemporary research in industrial/organizational psychology (Ashforth et al., 2008; Greco et al., 2022) notably due to its clear links to the Great Resignation^1^ (Klotz, 2022) and staff turnover (Riketta, 2005). Considered one of the main factors that can explain the investments and sacrifices employees are willing to make for their organization (Ashforth and Schinoff, 2016), OI is a predictor of a wide range of outcomes (Riketta, 2005; Lee et al., 2015; Greco et al., 2022), including: job involvement, job satisfaction, work engagement, and in-role and extra-role performance.

Inspired by the work of Mael and Ashforth (1992) on OI, Shamir and Kark (2004) created an instrument in English that has two distinguishing features: (1) it consists of a single item and (2) it is presented in a graphic form. On the one hand, single-item devices are appreciated for their simplicity and speed of administration (Fisher et al., 2016), sometimes offering an effective alternative to traditional multi-item instruments (Brown and Grice, 2011; Matthew et al., 2022), especially in work environments that are fast-paced, volatile and increasingly difficult to control (Ryan and Pulakos, 2007), i.e., asking researchers to adapt and speed up their assessment practices. In addition, these instruments reduce respondents’ cognitive load, arouse their interest, stimulate new thinking, minimize response bias, and may also promote higher participation rates (Allen et al., 2022). Although single-item instruments are sometimes criticized for their difficulty in translating complex multi-dimensional psychological concepts, recent work suggests that they may be as valid and reliable as unidimensional measures comprising multiple written items (see Allen et al., 2022; Fisher et al., 2016; Matthew et al., 2022).

On the other hand, graphical single-item instruments are a very special subform of such measurement tools. Notably, these instruments make it possible to measure constructs as varied as job satisfaction (Kunin, 1955), work intensity (Soucek and Voss, 2022), burnout (Muir et al., 2023), and energy level (Weigelt et al., 2022). Although not free of problems, including those related to cultural differences or the obsolescence of the elements presented graphically (Sauer et al., 2021), these instruments have the advantage of broadening the pool of potential respondents by including, for example, people with low literacy levels (Kunin, 1955; Sauer et al., 2021). Their use therefore makes it possible to broaden the range of respondent profiles that can participate in a study or respond to a request from an organization. Moreover, studies on the reliability and validity of these instruments show that they are generally less cognitively demanding on respondents, are completed faster, facilitate data collection that requires repeated measurements, and limit the impact on the cost of use (Allen et al., 2022; Matthew et al., 2022; Sauer et al., 2021).

Given the previously discussed advantages, the graphical single-item developed by Shamir and Kark (2004) seems an interesting and valid alternative for measuring OI, which also justifies the relevance of continuing the work initiated by Brousseau and Lauzier (2024) on its adaptation for French populations. While also responding to the call from Allen et al. (2022) for more work on the validation of single-item measures, this research has three objectives. First, it seeks to evaluate the level of readability of a French version of the graphic item of OI [adapted by Brousseau and Lauzier (2024)] compared to an instrument measuring the same construct, but which includes multiple written items. Second, this research aims to estimate the validity of the graphical single-item by showing, first, the existence of a positive correlation between the score observed on it and the score observed on an instrument measuring the same construct but which includes multiple written items; on this front, the research also shows an incremental contribution by the graphical single-item, i.e., beyond the variance explained by the instrument comprising multiple written items, in explaining certain criterion variables often studied in relation to OI (e.g., job satisfaction and work engagement). Third, this research aims to evaluate the reliability of the French version of the OI graphic item through a test-retest.

## Study 1

### Objectives

This first study aims to evaluate the level of readability of the French version of the graphic item of the OI translated by Brousseau and Lauzier (2024), as well as to determine the degree to which it converges with the instrument comprising multiple written items developed by Mael and Ashforth (1992). This objective is based on the work of Shamir and Kark (2004), who observed high correlations between the two instruments, ranging from *r* = 0.51 to *r* = 0.69. This first study also seeks to evaluate the incremental contribution of the graphic item in its relationship to job satisfaction and work commitment. As observed by meta-analyses (Greco et al., 2022; Lee et al., 2015; Riketta, 2005) as well as other recent reviews on the subject (Weisman et al., 2023), these constructs were chosen because of the repeated positive relationships they have with OI.

### Methodology

#### Participants

Participants in this study were drawn from two separate samples (*n*_1_ = 376; *n*_2_ = 80), mostly consisting of women (*n*_1_ = 301 women, 80%; *n*_2_ = 63 women, 78.8%), with mean ages (*M n*_1_ = 43.84 years [*SD* = 9.72]; *M n_2_* = 40.63 years [*SD* = 11.24]) and similar levels of work experience (*M n*_1_ = 8.63 years [*SD* = 7.83]; *M n*_2_ = 11.46 years [*SD* = 9.17]). Participants in each sample completed an electronic questionnaire that included the graphical single-item, the multi-item written instrument, and measures of job satisfaction and work engagement.

#### Instruments

##### Organizational identification (graphic instrument)

**FIGURE 1:**
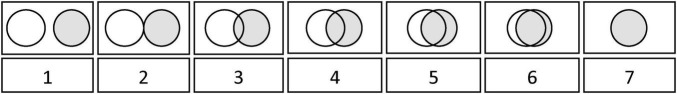
Graphical single-item of OI. Reproduce with permission from Shamir and Kark (2004), Journal of Occupational and Organizational Psychology, © 2004 The British Psychology Society. *French instructions* [from Brousseau and Lauzier (2024)]. Ce graphique a pour but d’évaluer votre relation avec l’organization à laquelle vous appartenez. Dans chacun des sept rectangles présentés ci-dessous, il y a deux cercles. L’un vous représente et l’autre représente votre organization. Dans le premier rectangle (numéro 1), ils sont totalement séparés et représentent une situation dans laquelle vous ne vous identifiez pas du tout à votre organization. Dans le dernier rectangle (numéro 7), les cercles sont totalement superposés et représentent une situation dans laquelle vous vous identifiez totalement à votre organization. Choisissez parmi les sept rectangles celui qui représente le mieux la mesure dans laquelle vous vous identifiez à votre organization. *English instructions* [from Shamir and Kark (2004)]. This chart is intended to assess your relationship with the organization you belong to. Above you will find seven rectangles. In each rectangle, there are two circles. One represents you and the other one the organization you belong to. In each rectangle, the circles overlap differently. In the first rectangle (number 1), they are totally separate and represent a situation in which you do not identify at all with your organization. In the last rectangle (number 7), the circles are totally overlapping and represent a situation in which you totally identify with your organization. Choose out of the seven rectangles the one that most highly represents the extent to which you identify with your organization.

The graphic single-item developed by Shamir and Kark (2004), then translated by Brousseau and Lauzier (2024), was one of the instruments used to measure OI. This graphical device comprises seven pairs of circles (Figure 1), each presenting a different degree of overlap between circles, ranging from zero (i.e., rectangle 1) to complete (i.e., rectangle 7). Greater overlap between the circles contained in each rectangle indicates a higher level of identification, whereas a less pronounced overlap between the circles indicates a lower level of OI.

##### Organizational identification (written instrument)

A French version of the instrument developed by Mael and Ashforth (1992) was used to measure this construct. Translated by Stinglhamber et al. (2015), this instrument consists of six items (e.g., When I talk about my organization, I usually say “we” rather than “they”) and uses a seven-point Likert scale to have respondents express their degree of agreement (1 = Strongly disagree; 7 = Strongly agree). A high score on this instrument indicates a higher level of identification with the organization. In this first study, Cronbach’s alphas of n_1_ = 0.902 and n_2_ = 0.936 are observed for this instrument.

##### Job satisfaction

Inspired by the work of Dolbier et al. (2005), this variable was measured using a written single-item (e.g., I am generally satisfied with my work) with a seven-point Likert scale (1 = Strongly disagree; 7 = Strongly agree). A high score on this item indicates a higher level of satisfaction. The valid reporting of job satisfaction using such an item has been recognized by numerous studies (Fisher et al., 2016; Tavani et al., 2014; Wanous et al., 1997).

##### Work engagement

This variable was measured using the instrument developed and translated by Schaufeli et al. (2006), which consists of 9 written items (e.g., I am full of energy for my work) and has a seven-point Likert scale (1 = Strongly disagree; 7 = Strongly agree). Scoring high on this instrument means a higher level of engagement. In this first study, Cronbach’s alphas of *n*_1_ = 0.922 and *n*_2_ = 0.925 are observed for this instrument.

### Results

The respective readability levels of the graphic and written measures of OI were obtained through *Scolarius*^2^, an online app that quantifies the extent to which a text in French is accessible. The principles and formulas that guide this app are widely used in the field of linguistics in general (see Pires et al., 2017), as well as when creating or adapting instruments intended for French populations (e.g., Robichaud et al., 2024). The smaller the index given the app, the more the text is readable and therefore accessible. The higher the index, the more difficult the text is considered to be and therefore preferable for specialized or more educated audiences. In this first study, the indices observed via the application show that the French version of the graphic item is more accessible (i.e., 115) than the French version of the multi-item written instrument developed by Mael and Ashforth (1992), which obtains an index suggesting a more difficult level of comprehensibility (i.e., 303).

As shown in Table 1, the results obtained in this first study also indicate that the scores observed from the graphic item and those from the written multi-item instrument correlate positively (*n*_1_ *r* = 0.619; *n*_2_ *r* = 0.654). For Sample 1, both instruments show positive correlations with job satisfaction (graphic item: *r* = 0.384; multi-item measure: *r* = 0.398) and work engagement (graphic item: *r* = 0.432; multi-item measure: *r* = 0.465). For Sample 2, both instruments show positive correlations with job satisfaction (graphic item: *r* = 0.548; multi-item measure: *r* = 0.654) and work engagement (graphic item: *r* = 0.516; multi-item measure: *r* = 0.568). Meng et al. (1992) provided a simple method for comparing (dependent) correlation coefficients. Based on the Fisher z-transform, this method makes it possible to estimate the differences between pairs of correlations coming from the same sample (e.g., by comparing the correlation of one predictor with one criterion compared to that of another predictor with the same criterion). As shown in Table 1, comparisons of correlation pairs (based on a dependent correlation test) show no statistically significant differences (i.e., *p* < 0.05). The absence of significant results for these few tests suggests that the strength of the relationship is statistically comparable between instruments. Finally, hierarchical regression analysis conducted on both samples in this first study shows that the results obtained from the graphic item present an improvement (see incremental contribution) in predicting each criterion variable (Table 2), i.e., beyond what the multi-item measure already predicts. This pattern held across all regression models except one for job satisfaction (Sample 2). Although marginally significant (*p* < 0.10), the effect is directional only and should be interpreted cautiously (Pritschet et al., 2016).

**TABLE 1:** Means, standard deviations and correlations between studied variables (study 1).

	Sample 1: *N* = 376					Sample 2: *N* = 80				
Variables	M	SD	*r* ^(1)^	*r* ^(2)^	*r* ^(3)^	M	SD	*r* ^(1)^	*r* ^(2)^	*r* ^(3)^
Descriptive statistics and correlations between instrument types										
OI (graphic single-item)	3.989	1.346	0.619**			4.550	1.466	0.654**		
OI (written multi-items)	4.836	1.313				5.506	1.288			
Descriptive statistics and correlations with the criterion variables										
Job satisfaction	5.547	1.321		0.384**	0.398**	5.812	1.264		0.548**	0.516**
Work engagement	5.223	1.137		0.432**	0.465**	5.515	1.066		0.654**	0.568**

Gender: 1 = Male; 0 = Female; M = Mean; SD = Standard deviation; *r*^(1)^ = Correlations between instruments (graphical – multi-items); *r*^(2)^ = Correlations with the graphical instrument; *r*^(3)^ = Correlations with the multi-item instrument.

**p < 0.01.

**TABLE 2:** Incremental contribution of the OI graphic single item on job satisfaction and work engagement (study 1).

	Sample 1: *N* = 376						Sample 2: *N* = 80					
	Job satisfaction			Work engagement			Job satisfaction			Work engagement		
Variables	S1	S2	S3	S1	S2	S3	S1	S2	S3	S1	S2	S3
Gender	0.059	0.034	0.041	0.076	0.047	0.055	−0.213^†^	−0.145	−0.107	−0.115	−0.058	−0.006
Age	−0.004	−0.024	−0.030	0.031	0.008	0.003	0.049	0.066	−0.060	0.321*	0.223^†^	0.231*
Tenure	0.057	0.062	0.043	0.074	0.086	0.067	0.157	0.138	0.131	−0.095	−0.112	−0.120
OI (multi-items)		0.396**	0.259**		0.460**	320**		0.617**	0.496**		30**	0.369**
OI (graphic item)			0.221**			0.226**			0.191^†^			0.255**
*R2*	0.007	0.163	0.192	0.016	0.226	0.247	0.078	0.437	0.457	0.093	0.358	0.393
*ΔR2*		0.156	0.030		0.210	0.031		0.359	0.019		0.265	0.035

S1 = Step 1 (socio-demographic indices); S2 = Step 2 (multi-items instrument); S3 = Step 3 (graphic item). OI multi-items = Instrument developed by Mael and Ashforth (1992); OI graphic item = Single graphical item developed by Shamir and Kark (2004).

**p < 0.01;

*p < 0.05;

† *p* < 0.10.

### Discussion

Overall, the results of this first study show that the French version of the graphic item compares adequately to its counterpart comprising multiple written items. Notably, the graphic item has a better readability index than that observed for the other instrument, which favors broader usability, including that for workers with lower literacy levels. In terms of convergent validity, the results of this first study are similar to those observed by Shamir and Kark (2004). Finally, the results also show the incremental contribution of the graphic item in explaining job satisfaction and work engagement.

## Study 2

### Objectives

In addition to evaluating the reliability (test-retest) of the French adaptation of the single graphic item, the second study aims to replicate the results observed in the first. Test–retest reliability is crucial for single items as it ensures they produce consistent results over time. In this respect, Shamir and Kark (2004) reported high test-retest correlations (based on a 2-weeks interval between tests), ranging from *r* = 0.73 to *r* = 0.80 for two of their samples. Brousseau and Lauzier (2024), on the other hand, observed a test-retest correlation equivalent to *r* = 0.66 [ICC = 0.69] established according to a 3-months interval between test administrations. For this second study, a 1-month interval between test administrations was preferred (Vallerand, 1989).

### Methodology

#### Participants

This second study relied on a convenience sampling method. The survey group consisted of 92 Canadian respondents who completed a brief questionnaire twice, containing the graphic item. Respondents in this second study were on average 25.88 years old (*SD* = 7.72), were a little over half women (55; 59.8%) and had an average of 3.19 years of experience (*SD* = 3.55) in the job held at the time of the study.

#### Instruments

All variables were measured with the instruments used for Study 1.

### Results

First, the calculation of the correlation between the scores obtained with each instrument reveals some convergence (*r* = 0.540). Although within the range of results observed by Shamir and Kark (2004), this result is somewhat lower than the correlations found by the first study. Again, using Meng et al.’s (1992) method for comparing pairs of (dependent) correlations did not indicate a significant difference between instruments used (i.e., *p* < 0.05). Hierarchical regression analyses conducted on this new sample also show the incremental contribution of the single graphic item in explaining job satisfaction and work engagement (see Table 3).

**TABLE 3:** Incremental contribution of the OI graphic single item on job satisfaction and work engagement (study 2).

	Sample 3: N = 92					
	Job satisfaction			Work engagement		
Variables	S1	S2	S3	S1	S2	S3
Gender	−0.076	−0.053	−0.050	−0.198	−0.168	−0.165*
Age	−0.119	−0.139	−0.129	0.193	0.167	0.178*
Tenure	0.107	0.107	0.098	−0.073	−0.074	−0.085
IO (multi-items)		0.428**	0.294**		0.560**	0.407**
IO (graphic item)			0.264*			0.302**
*R2*	0.023	0.205	0.257	0.070	0.382	0.450
*ΔR2*		0.182	0.052		0.312	0.068

S1 = Step 1 (socio-demographic indices); S2 = Step 2 (multi-item instrument); S3 = Step 3 (graphic item). OI multi-items = Instrument developed by Mael and Ashforth (1992); OI graphic item = Single graphical item developed by Shamir and Kark (2004).

**p < 0.01;

*p < 0.05.

While the calculation of a Pearson *r* is the most common approach for estimating test-retest reliability, Matthew et al. (2022) indicate that this procedure offers little guidance as to what constitutes “acceptable” reliability. Thus, some suggest that it is preferable to calculate an intra-class correlation coefficient (ICC), considering that this index offers benchmarks (i.e., ICC closer to 0 indicates low/poor reliability and ICC closer to 1 indicates high/excellent reliability). Hence, to evaluate the graphic item’s test-retest reliability, Pearson *r* and ICC were calculated between the scores provided by the respondents at each measurement time. According to Cicchetti (1994), ICC values greater than 0.74 indicate excellent reliability, those between 0.60 and 0.74 good reliability, those between 0.40 and 0.59 medium reliability, and those below 0.40 low reliability. In this second study, a positive correlation was found (*r* = 0.818; ICC = 0.815). Above all, these results suggest that the two ways of estimating the test-retest reliability of the graphical item (i.e., Pearson *r* and ICC) offer fairly similar values, a finding that is consistent with the observations made by Matthew et al. (2022).

### Discussion

Overall, the observations made in this second study suggest that the French adaptation of the graphic single-item converges with a French version of the instrument comprising multiple written items developed by Mael and Ashforth (1992), while reconfirming its incremental contribution in explaining job satisfaction and work engagement. Also, the results of this new study show excellent test-retest reliability for this French version of the graphic item. In this respect, it is relevant to note that the values reported by Shamir and Kark (2004), despite a shorter interval between test administrations (i.e., only 2 weeks), are lower than those observed in this second study.

## General discussion

The main objective of this research was to further advance the psychometric qualities of a French version of a graphic item of OI. Overall, the results indicate a convergence between the graphic single item and an instrument with multiple written items, also measuring OI. This validation is based on analyses conducted on three distinct samples, collected in different contexts from various work environments. Although there may be slight variations in each study’s findings, it is reassuring that the relationships and indices observed are generally in line with previous work. Taken together, the results of this study, when compared to those of Shamir and Kark (2004) and those of Brousseau and Lauzier (2024), are considered satisfactory.

### Limitations of the study and future avenues of research

The validation of any measuring instrument is an ongoing process. Although the convergent validity of this French adaptation of the graphic single-item of OI is evident and its incremental contribution now recognized, the results observed in these two studies suggest that the two instruments (graphic and written) can probably not be considered as simple substitutes. Hence, the qualities of the graphic single-item deserve further exploration in the context of future research. In particular, this work could allow for a more precise documentation of the general functioning of the graphic single-item by examining in greater detail some of the slight inter-sample variations observed in the two studies. Also, the generalizability of these findings may be limited by the overrepresentation of women in Study 1 and the relatively young, low-tenure profile of participants in Study 2. Hence, future research should aim to replicate these results with more diverse and representative samples.

Regarding the measurement of the criterion variables used in this study, it is important to note that it is based on respondents’ perceptions only. Given the biases commonly associated with self-reported measures (Podsakoff et al., 2024), the use of objective measures (such as effective turnover rate) represents a promising avenue for further efforts to validate this French adaptation of the graphic single-item. In addition, a major challenge that researchers face in this field is the organization of data collections, including different measurement points in time or coming from multiple sources. Given its brevity, the graphic item could facilitate the collection of dyadic data (i.e., by shortening the time each dyad member needs to respond), thus offering a more nuanced view of French-speaking workers’ OI level. Another promising avenue for future research lies in the evolutionary approach that characterizes OI. Indeed, some changes can induce significant transformations over time in terms of potentially influencing workers’ attitudes and, consequently, their level of identification with their organization (Stinglhamber et al., 2015). Further studies are therefore needed to better understand the evolution of OI as measured by this graphic item among French-speaking worker populations. Coming research could also investigate whether the graphic items capture more intuitive and/or affective components of organizational identification (OI) compared with traditional measures.

## Conclusion

The objective of this research was to further substantiate the psychometric qualities of a graphic single-item of OI translated into French by Brousseau and Lauzier (2024). More specifically, this research has shown through two studies the convergent validity of the graphic item with its counterpart comprising multiple written items, while also showing its incremental contribution to explain certain criterion variables commonly studied in relation to OI (i.e., job satisfaction and work engagement). Finally, this study also validates the excellent test-retest reliability of the French adaptation of the graphic item. Short, effective and accessible, this version appears to be a useful and attractive alternative for collecting data from Francophone worker populations.

## Data Availability

The datasets generated for this study are available upon reasonable request to the corresponding author.

## Footnotes

1 The Great Resignation (or Great Reshuffle), a term coined by Klotz (2022), describes a post-2021 trend where large numbers of workers chose to leave their jobs in search of higher pay, greater flexibility, improved work-life balance, and better mental health, leading to workforce shortages in many sectors and forcing employers to adapt.

2
https://www.scolarius.com
